# *Xanthoceras sorbifolium* Bunge Leaves Ameliorate Type 2 Diabetes Mellitus by Modulating Glucolipid Metabolism Through the Gut Microbiota–Metabolite Axis

**DOI:** 10.3390/foods14223809

**Published:** 2025-11-07

**Authors:** Qiong Jia, Xianyu Zhang, Mengting Han, Tian Zhong, Hui Zhou

**Affiliations:** 1 Key Laboratory of Biotechnology and Bioresource Utilization, Ministry of Education, Institute of Plant Resources, Dalian Minzu University, Dalian 116600, China; jiaqiong2277@163.com (Q.J.); zhangxianyu200110@163.com (X.Z.); hanmengting126@163.com (M.H.); 2 Faculty of Medicine, Macau University of Science and Technology, Taipa, Macao 999078, China

**Keywords:** *Xanthoceras sorbifolium* Bunge leaves, gut microbiota modulation, fecal metabolomics, short-chain fatty acids, multi-omics approach, natural functional food

## Abstract

*Xanthoceras sorbifolium* Bunge leaves (XBL), traditionally consumed as herbal tea, have attracted increasing attention as potential functional food ingredients for managing type 2 diabetes mellitus (T2DM). This study investigated the anti-diabetic effects of an aqueous XBL extract in T2DM rats induced with a high-fat, high-sucrose diet combined with streptozotocin. XBL administration significantly improved glycemic control, insulin sensitivity, lipid profiles, and pancreatic and renal histopathology. Integrated 16S rRNA sequencing and untargeted fecal metabolomics revealed the modulation of key metabolic pathways, including linoleic acid and histidine metabolism, and elevated production of short-chain fatty acids (SCFAs) such as acetate and propionate. XBL also enriched beneficial gut microbes including *Prevotella*, *Lachnospiraceae_NK4A136_group*, and *[Eubacterium]_xylanophilum_group*, whose abundance showed positive correlations with SCFA levels and metabolic improvements. These findings demonstrate that XBL ameliorates T2DM through gut microbiota–SCFA–metabolite interactions and suggest its potential as a natural, multi-target dietary strategy for metabolic health management.

## 1. Introduction

Type 2 diabetes mellitus (T2DM) is a chronic metabolic disorder marked by persistent hyperglycemia, primarily resulting from insulin resistance and β-cell dysfunction [1]. The International Diabetes Federation projects that over 780 million individuals will be affected by diabetes by 2045 [2,3], highlighting the urgent need for safe, effective, and sustainable treatment strategies. Uncontrolled T2DM is closely linked to multiple complications, including nephropathy, cardiovascular disease, and stroke [4,5,6]. While pharmacological agents such as metformin and GLP-1 analogs remain mainstays of treatment [7], their limited efficacy and adverse effects, including gastrointestinal symptoms, hypoglycemia, and weight gain, may compromise long-term compliance [8]. These limitations have prompted increasing interest in alternative and adjunctive therapies, particularly those derived from natural sources. Functional foods rich in bioactive compounds offer promising multi-target interventions for metabolic regulation and disease prevention.

Recent advances have identified the gut microbiota as a central player in metabolic homeostasis and a promising target for T2DM therapy [9]. This dynamic and responsive microbial ecosystem is influenced by host factors such as age, diet, and medication, and plays a critical role in shaping metabolic outcomes through its metabolites [10]. Dysbiosis—characterized by the loss of microbial diversity and enrichment of pro-inflammatory species—has been associated with impaired intestinal barrier function, reduced production of short-chain fatty acids (SCFAs), and systemic inflammation [11,12,13]. SCFAs, especially acetate, propionate, and butyrate, contribute to glycemic control by regulating energy metabolism, enhancing insulin secretion, and modulating endocrine pathways. These insights have fueled the development of microbiota-targeted interventions, including probiotics, fecal microbiota transplantation, and plant-derived functional foods, aiming to restore microbial balance and improve host metabolism [9,14].

*Xanthoceras sorbifolium* Bunge, a deciduous tree native to northern China and a member of the Sapindaceae family, has been used in both culinary and medicinal contexts [15]. *Xanthoceras sorbifolium* Bunge leaves (XBL), often discarded as industrial by-products, are rich in bioactive constituents such as flavonoids, polyphenols, alkaloids, and saponins. These compounds exhibit diverse biological activities, including antioxidant, anti-inflammatory, lipid-lowering, and hypoglycemic effects, which are closely associated with the metabolic disturbances underlying T2DM. Although such properties imply potential benefits for glucose and lipid regulation, the direct anti-diabetic efficacy and underlying mechanisms of XBL have not yet been systematically investigated [16,17,18,19,20]. Despite these observations, most studies have focused on ethanol-based extracts, while the aqueous extract, which is more representative of traditional consumption and more suitable for functional food development, remains underexplored. Furthermore, the potential role of XBL in modulating gut microbiota composition and fecal metabolite profiles in T2DM has not been systematically examined.

To address this gap, the present study investigated the anti-diabetic efficacy of aqueous XBL extract using a validated rat model of T2DM induced via dietary and chemical stress. A multi-omics approach integrating 16S rRNA gene sequencing and untargeted fecal metabolomics via UHPLC-Q-TOF-MS was employed to dissect the association between microbial composition, SCFA production, and host metabolism. Given the complex interplay between the gut microbiota, their metabolites, and host metabolism, this study employed an integrated multi-omics approach that combined 16S rRNA gene sequencing and untargeted fecal metabolomics via UHPLC-Q-TOF-MS to comprehensively elucidate the mechanisms by which XBL exerts its anti-diabetic effects. This combined analysis provides a systems-level perspective that links microbial community dynamics with metabolic outcomes, offering mechanistic insights that cannot be obtained through conventional single-omics or biochemical approaches. By focusing on the gut microbiota–SCFA–metabolite axis, this study aims to elucidate the mechanistic basis of XBL’s therapeutic effects. The findings not only expand our understanding of plant-based interventions for metabolic diseases but also highlight XBL as a high-value candidate for developing functional foods targeting T2DM and associated glucolipid imbalances.

## 2. Materials and Methods

### 2.1. Materials and Reagents

Streptozotocin (STZ) was purchased from Sigma-Aldrich (St. Louis, MO, USA). Biochemical assay kits for triglycerides (TG; Cat. No. 20241014) and total cholesterol (TC; Cat. No. 20241010) were obtained from the Jiancheng Bioengineering Institute (Nanjing, China). A glucometer and test strips were supplied by Yicheng Bioelectronic Technology Co. (Beijing, China). HPLC-grade methanol, acetonitrile, and formic acid were sourced from Fisher Scientific (Waltham, MA, USA). Ultrapure water was produced using a Milli-Q purification system (Millipore, Billerica, MA, USA).

### 2.2. Preparation of XBL Extract

The aqueous extract of XBL was provided by Ningxia Vanilla Biotechnology Co., Ltd. (Ningxia, China). The raw plant material was harvested from the Daxing’anling and Xiaoxing’anling regions in northeastern China. Dried leaves were extracted in distilled water at a 1:10 (*w*/*v*) ratio under reflux for 1 h, and the procedure was repeated twice. The combined extracts were filtered, concentrated under reduced pressure, and spray-dried to obtain a powdered product. A voucher specimen (No. 20230925) was deposited at the Institute of Plant Resources, Dalian Minzu University. The extract was stored at −20 °C until further use.

### 2.3. Animal Experiment and Study Design

Male Sprague-Dawley (SD) rats (160–200 g) were purchased from Liaoning Changsheng Biotechnology Co., Ltd. (Liaoning, China). Animals were housed under controlled environmental conditions (23 ± 2 °C, 50% ± 5% humidity, 12 h light/dark cycle) with free access to food and water. After 1-week of acclimatization, the animals were divided into control and experimental groups. The control group received standard chow, while the other rats were fed a high-fat, high-sucrose diet (HFD) for 8 weeks to induce metabolic dysfunction. Subsequently, a single intraperitoneal injection of STZ (35 mg/kg) was administered. Rats with fasting blood glucose (FBG) levels > 16.7 mmol/L one week post-injection were considered diabetic [21].

**Figure 1:**
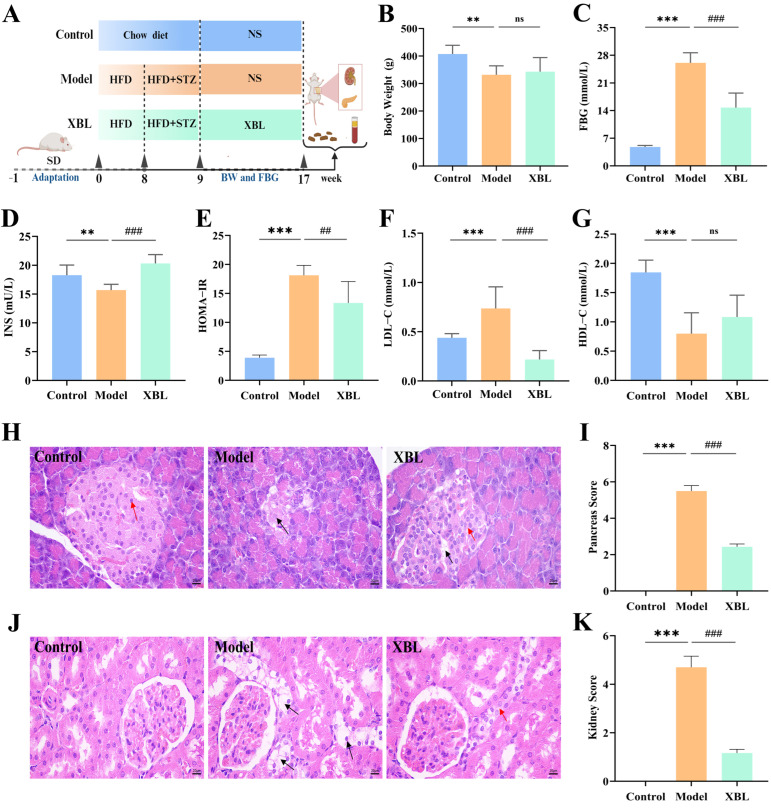
Effects of XBL treatment on metabolic and histopathological parameters in T2DM rats. (**A**) Schematic diagram of the experimental design for XBL intervention in HFD and STZ-induced T2DM rats. All biochemical parameters and histopathological evaluations were assessed after eight weeks of XBL treatment. (**B**) BW. (**C**) FBG levels. (**D**) INS concentrations. (**E**) HOMA-IR index. (**F**) LDL-C levels. (**G**) HDL-C levels. (**H**) Representative images of H&E-stained pancreatic tissue sections (400× magnification; scale bar = 20 μm). Red arrows indicate pancreatic islet cells; black arrows indicate cytoplasmic vacuolization. (**I**) Histopathological scores of pancreatic tissues. (**J**) Representative H&E-stained kidney tissue sections (400× magnification; scale bar = 20 μm). Red arrows indicate tubular epithelial cells; black arrows indicate cytoplasmic vacuolization. (**K**) Histopathological scores of kidney tissues. Data are presented as mean ± SEM (*n* = 8 per group). Statistical analysis was performed using one-way ANOVA followed by Tukey’s post hoc test. ** *p* < 0.01, *** *p* < 0.001 vs. Control group; ^##^ *p* < 0.01, ^###^ *p* < 0.001 vs. Model group.

Diabetic rats were randomized into two groups: a T2DM model group and an XBL treatment group. The treatment group received oral gavage of XBL at 2 g/kg/day for 8 weeks. XBL was freshly dissolved in ultra-pure water before administration. To ensure comparable handling conditions, both the control and model groups were administered an equal volume of the vehicle (ultra-pure water) via oral gavage on the same schedule. The dosage was based on prior research and refined through pilot studies. A schematic overview of the experimental protocol is provided in Figure 1A.

### 2.4. Biochemical Analysis

Body weight (BW) and fasting blood glucose (FBG) were measured weekly throughout the intervention. Blood samples were collected from the tail vein following a 12 h fast. FBG was measured using a glucometer (Roche, Suzhou, China) following the manufacturer’s instructions. Serum was isolated for the determination of fasting insulin (INS), low-density lipoprotein cholesterol (LDL-C), and high-density lipoprotein cholesterol (HDL-C). Insulin resistance was evaluated using the homeostasis model assessment index (HOMA-IR), calculated as:

$$HOMA-IR=\frac{FBG\;\left(mmol/L\right)\times \;FINS\;\left(\mu L/L\right)}{22.5}$$

### 2.5. Histopathological Evaluation

At the end of the 8-week intervention, the pancreas and kidney were excised, rinsed in cold saline, and fixed in 10% neutral-buffered formalin. After dehydration and paraffin embedding, tissue sections (4–5 μm) were cut and stained with hematoxylin and eosin (H&E). Histological changes were evaluated using light microscopy.

### 2.6. Fecal Metabolomics

Fecal samples were collected using metabolic cages and stored at −80 °C. For extraction, samples were mixed with chilled methanol:water (4:1, *v*/*v*) at a 1:10 (*w*/*v*) ratio, followed by homogenization using ball milling and 10 min of ultrasonic treatment in an ice bath. The homogenate was centrifuged at 12,000 rpm for 10 min at 4 °C, and the supernatant was used for LC–MS analysis.

Chromatographic separation was conducted on a Waters ACQUITY UPLC I-Class Plus system equipped with an HSS T3 C18 column (100 mm × 2.1 mm, 1.8 μm). The mobile phases were 0.1% formic acid in water (A) and acetonitrile (B), with a flow rate of 0.35 mL/min. The gradient program was as follows: 0–2 min, 5% B; 2–4 min, 30% B; 4–8 min, 50% B; 8–10 min, 80% B; and 10–14 min, 100% B.

Mass spectrometry was performed using a Q Exactive mass spectrometer (Thermo Scientific, Waltham, MA, USA) with electrospray ionization (ESI). The spray voltages were 3.8 kV (positive mode) and 3.0 kV (negative mode). Other parameters included the following: capillary temperature, 320 °C; sheath gas, 35 arbitrary units; auxiliary gas, 8 units; scan range, *m*/*z* 70–1050; and stepped normalized collision energies (NCE) of 10, 20, and 40.

### 2.7. 16S rRNA Gene Sequencing

Genomic DNA was extracted from fecal samples using the MagPure Soil DNA LQ Kit (Cat. No. D6356-02, Magen,  Guangzhou, China). The V3–V4 hypervariable regions of the 16S rRNA gene were amplified using primers 343F (5′-TACGGRAGGCAGCAG-3′) and 798R (5′-AGGGTATCTAATCCT-3′). Amplicon libraries were constructed and sequenced on a NovaSeq platform, producing 250 bp paired-end reads.

Raw sequencing data were processed using QIIME2. Quality filtering, denoising, chimera removal, and feature table construction were carried out using the DADA2 plugin. Taxonomic classification was performed using the SILVA 138 database and the classify-sklearn method. Alpha diversity was assessed with the ACE index; beta diversity was evaluated using PCoA based on Bray–Curtis and weighted UniFrac distances. LEfSe analysis (LDA > 3.0) was used to identify differentially abundant taxa.

### 2.8. Short-Chain Fatty Acid (SCFA) Profiling

Approximately 30 mg of fecal material was extracted with 300 μL of acetonitrile containing internal standards [^2^H_9_]-pentanoic acid and [^2^H_11_]-hexanoic acid. After bead homogenization and ultrasonic treatment, the samples were centrifuged at 12,000 rpm for 10 min at 4 °C. Supernatants were diluted 1:5 and derivatized by reacting with equal volumes of 200 mM 3-nitrophenylhydrazine (3-NPH) and 120 mM EDC in 6% pyridine at 40 °C for 30 min, followed by cooling on ice.

The derivatized samples were analyzed using a Waters ACQUITY H-Class UPLC with a BEH C18 column (100 mm × 2.1 mm, 1.7 μm). Mobile phases consisted of 0.1% formic acid in water (A) and a mixture of acetonitrile:methanol (2:1, *v*/*v*) as solvent B. The gradient was: 0–2 min, 25% B; 2–11 min, 55% B; 11–13 min, re-equilibration at 25% B. The flow rate, injection volume, and column temperature were 0.35 mL/min, 1 μL, and 40 °C, respectively.

Detection was performed using a QTRAP 5500 system (SCIEX, Boston, MA, USA) in negative ESI mode at −4500 V. The ion source temperature was 450 °C, with curtain gas at 35 psi and both Gas1 and Gas2 at 50 psi. Nitrogen served as the carrier and collision gas. Data were analyzed using Analyst and SCIEX OS-MQ software 3.4.5.

### 2.9. Statistical Analysis

**Figure 2:**
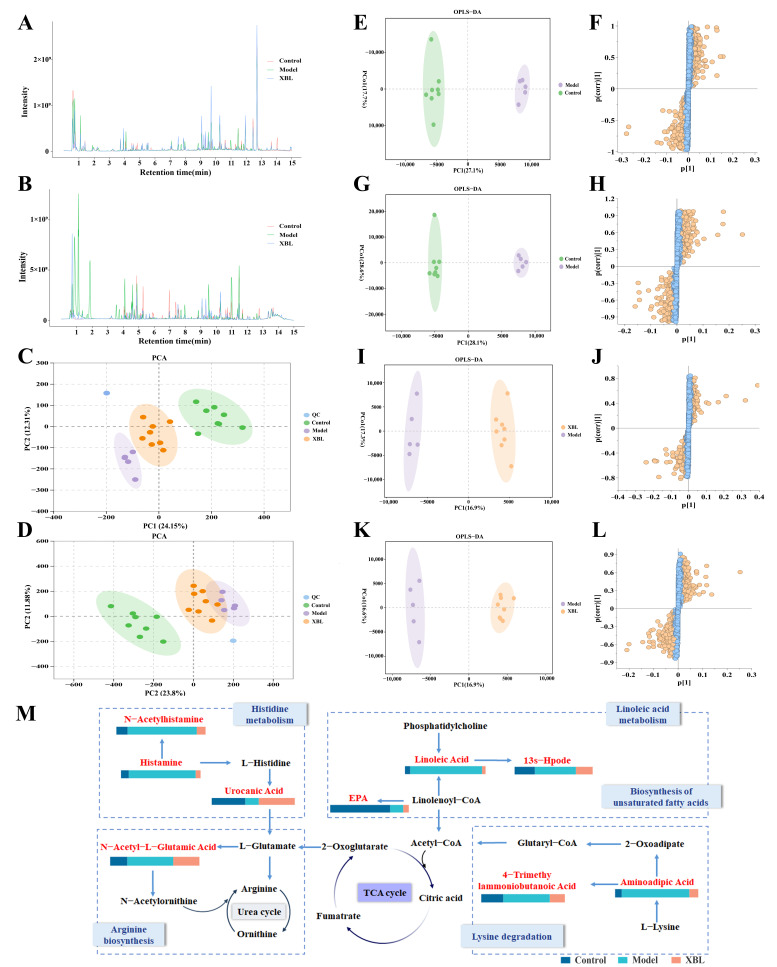
Metabolic profiling and pathway analysis of endogenous metabolites in fecal samples from T2DM rats after XBL intervention. (**A**,**B**) Base peak chromatograms (BPC) of fecal metabolites in positive and negative ion modes. (**C**,**D**) Principal component analysis (PCA) score plots in positive and negative ion modes. (**E**,**F**) OPLS-DA score plot and corresponding S-plot comparing the Control and Model groups in positive ion mode. (**G**,**H**) OPLS-DA score plot and corresponding S-plot comparing the Control and Model groups in negative ion mode. (**I**,**J**) OPLS-DA score plot and corresponding S-plot comparing the Model and XBL groups in positive ion mode. (**K**,**L**) OPLS-DA score plot and corresponding S-plot comparing the Model and XBL groups in negative ion mode. (**M**) Schematic diagram of major metabolic pathways associated with potential biomarkers modulated by XBL intervention. Color bars represent 100% stacked responses of therapeutic biomarkers for each group (Control, Model, and XBL). Data are presented as mean ± standard deviation (SD) (*n* = 8 per group). For PCA and OPLS-DA visualizations (**C**–**L**), three samples were excluded from the Model group, and five samples were used for plotting.

Data are expressed as mean ± standard deviation (SD). Intergroup comparisons were performed using one-way ANOVA followed by Tukey’s post hoc test. Statistical significance was defined as *p* < 0.05. All graphs and statistical analyses were conducted using GraphPad Prism 10 (GraphPad Software, San Diego, CA, USA). Outliers were identified prior to multivariate analysis. For improved visualization in PCA and OPLS-DA plots (Figure 2C–L), three outliers from the model group were excluded, resulting in *n* = 8 for the control and XBL groups and *n* = 5 for the model group. However, full datasets (*n* = 8 per group) were used for all downstream analyses, including metabolite identification and pathway enrichment. Spearman’s correlation analysis was conducted to explore associations among fecal metabolites, microbiota composition, SCFAs, and metabolic indicators (BW, FBG, INS, LDL-C, HDL-C). Correlations were considered meaningful at *p* < 0.05 and |*r*| > 0.6.

## 3. Results

### 3.1. Pharmacodynamic Effects of XBL on Diabetic Rats

To evaluate the antihyperglycemic and metabolic regulatory properties of XBL, a T2DM model was established in rats via high-fat, high-sucrose feeding followed by low-dose STZ injection (Figure 1A). After eight weeks of intervention, the model group exhibited a significant decline in body weight (331.9 ± 32.52 g) compared to healthy controls (407.5 ± 31.62 g, *p* < 0.01). XBL treatment moderately improved body weight (352.3 ± 26.31 g), although the increase did not reach statistical significance relative to the untreated diabetic group (Figure 1B). In addition, food and water intake were recorded daily throughout the 8-week administration period. The diabetic rats exhibited markedly higher food and water intake compared with the control group, whereas XBL treatment alleviated these elevations, suggesting improvement in hyperphagia and polydipsia symptoms.

To assess glycemic regulation, FBG, INS, and HOMA-IR were measured. As shown in Figure 1C, FBG levels were markedly elevated in diabetic rats (26.08 ± 2.56 mmol/L) relative to the controls (4.80 ± 0.36 mmol/L, *p* < 0.001). XBL significantly reduced FBG to 14.76 ± 3.68 mmol/L (*p* < 0.001 vs. model). Insulin levels, which were suppressed in diabetic rats (15.68 ± 1.01 mU/L), showed substantial recovery following XBL administration (20.30 ± 1.54 mU/L, *p* < 0.001; Figure 1D). Correspondingly, HOMA-IR was significantly increased in the model group (18.14 ± 1.71) versus controls (3.90 ± 0.47, *p* < 0.001), and this elevation was significantly mitigated by XBL treatment (13.35 ± 3.71, *p* < 0.01; Figure 1E).

Dyslipidemia, a hallmark of metabolic syndrome, was also evident in the model group, which showed elevated LDL-C (0.74 ± 0.22 mmol/L) and reduced HDL-C (0.80 ± 0.36 mmol/L). XBL significantly reduced LDL-C to 0.22 ± 0.09 mmol/L (*p* < 0.001; Figure 1F). While the increase in HDL-C following XBL treatment (1.09 ± 0.37 mmol/L) did not reach significance, a clear upward trend was observed (Figure 1G).

To investigate the protective effects of XBL on diabetes-induced organ damage, histopathological analysis was performed. Pancreatic tissue from the model group revealed severe islet atrophy, vacuolation, and β-cell loss. In contrast, XBL treatment preserved islet architecture, restored cellular morphology, and reduced vacuolar degeneration (Figure 1H). The corresponding pancreatic pathology score declined significantly from 5.50 ± 0.30 in the model group to 2.43 ± 0.15 following XBL administration (*p* < 0.001; Figure 1I). Similarly, renal histology revealed pronounced tubular dilation, epithelial cell detachment, and vacuolar degeneration in diabetic rats (Figure 1J), which were markedly ameliorated with XBL, as reflected through a reduced renal pathology score (4.70 ± 0.46 to 1.17 ± 0.15, *p* < 0.001; Figure 1K).

Collectively, these findings demonstrate that XBL exerts multifaceted therapeutic effects in diabetic rats, including significant improvements in glycemic control, insulin sensitivity, lipid homeostasis, and histological integrity of pancreatic and renal tissues.

### 3.2. XBL Remodels the Fecal Metabolome and Modulates Key Metabolic Pathways

To investigate the metabolic mechanisms underlying the therapeutic effects of XBL, untargeted fecal metabolomics were performed. Base peak chromatograms acquired in both positive and negative electrospray ionization (ESI) modes exhibited consistent retention times and signal intensities across all groups, confirming the analytical stability and reproducibility of the LC–MS platform (Figure 2A,B).

PCA revealed a clear separation between the control and model groups, indicating profound metabolic perturbations induced by T2DM. Notably, the XBL-treated group formed a distinct cluster between the two, suggesting a partial restoration of the metabolic profile toward the control state. Furthermore, the tight clustering of QC samples demonstrated excellent analytical reproducibility, confirming the stability and reliability of the metabolomics data (Figure 2C,D). Orthogonal partial least squares discriminant analysis (OPLS-DA) further validated the separation, with excellent model performance metrics underscoring the robustness and predictive power of the models (Figure 2E,L): R^2^Y = 0.99 and Q^2^ = 0.93 (positive mode); R^2^Y = 0.99 and Q^2^ = 0.78 (negative mode).

To identify discriminative metabolites contributing to group differences, S-plot analysis was performed. Metabolites with variable importance in projection (VIP) scores > 1.0 and *p* < 0.05 were considered significant. A total of nine differential metabolites were annotated via MS/MS fragmentation and matched against the Human Metabolome Database (HMDB) (Table 1). In the diabetic state, the levels of 13s-hydroperoxyoctadecadienoic acid (13s-Hpode), linoleic acid, 4-trimethylammoniobutanoic acid, histamine, urocanic acid, and aminoadipic acid were significantly elevated. XBL treatment substantially attenuated these increases. Conversely, N-acetylhistamine, which was suppressed in diabetic rats, showed a restorative trend following XBL administration. Interestingly, eicosapentaenoic acid (EPA) remained low in both the model and XBL groups, suggesting that this lipid perturbation may not be directly targeted by XBL.

To contextualize these metabolic shifts, Kyoto Encyclopedia of Genes and Genomes (KEGG) pathway enrichment analysis was conducted. The bar chart in Figure 2M illustrates the relative abundance of key metabolites across groups. The most significantly affected pathways included histidine metabolism, arginine biosynthesis, lysine degradation, linoleic acid metabolism, and the biosynthesis of unsaturated fatty acids. Importantly, metabolites such as linoleic acid, histamine, N-acetylhistamine, urocanic acid, and aminoadipic acid were functionally linked to these pathways, highlighting XBL’s regulatory role in amino acid and lipid metabolism. The reversal of linoleic acid-derived lipid mediators and histidine catabolites suggests a potential anti-inflammatory mechanism mediated via gut microbial and host co-metabolism.

Together, these metabolomic findings reveal that XBL profoundly reshapes the fecal metabolic landscape in T2DM, targeting specific pathways involved in fatty acid oxidation, amino acid catabolism, and host–microbiota interactions.

### 3.3. XBL Modulates Gut Microbial Dysbiosis in T2DM Rats

Given the central role of gut microbiota in host metabolic homeostasis, we then assessed whether the metabolic benefits of XBL were accompanied by compositional shifts in the intestinal microbiota. To this end, 16S rRNA gene sequencing was performed on fecal samples from the control, model, and XBL-treated T2DM groups.

**Figure 3:**
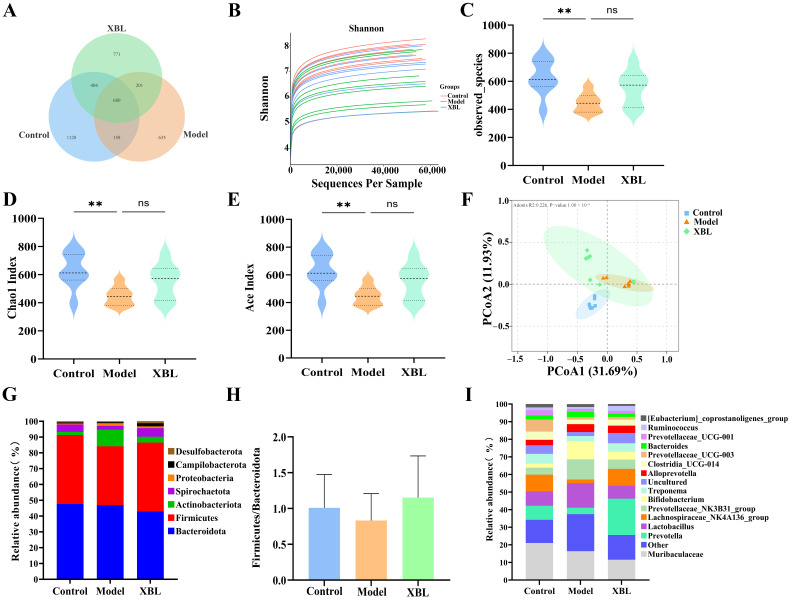
Effects of XBL treatment on gut microbiota diversity and composition in T2DM rats. (**A**) Venn diagram showing the shared and unique amplicon sequence variants (ASVs) among the Control, Model, and XBL groups. (**B**) Shannon rarefaction curves evaluating sequencing depth and microbial diversity sufficiency. (**C**–**E**) α-Diversity indices, including observed species richness, Chao1 index, and ACE index. (**F**) β-Diversity visualized by principal coordinates analysis (PCoA) based on unweighted Bray–Curtis distance metrics. (**G**) Relative abundance of gut microbiota at the phylum level. (**H**) *Firmicutes*/*Bacteroidota* ratio among groups. (**I**) Relative abundance of gut microbiota at the genus level. Data are presented as mean ± standard deviation (SD) (*n* = 8 per group). Statistical significance: ** *p* < 0.01.

An ASV-based Venn diagram revealed that 680 ASVs were shared across all groups, while 1128, 635, and 771 ASVs were unique to the control, model, and XBL groups, respectively (Figure 3A), suggesting that T2DM and XBL intervention both exerted substantial influences on microbial composition. Sequencing depth and community diversity were confirmed by the platform sparsity and Shannon rarefaction curves (Figure 3B). Alpha diversity analysis showed significant reductions in observed species, Chao1, and ACE indices in the model group, indicating impaired microbial richness and evenness. While XBL did not fully normalize these metrics, it induced a partial but meaningful restoration of microbial diversity (Figure 3C–E).

Principal coordinate analysis (PCoA) based on Bray–Curtis distances revealed distinct clustering patterns across groups. The gut microbiota of the model group deviated markedly from that of healthy controls, reflecting diabetes-associated dysbiosis. Notably, XBL treatment elicited a distinct shift in microbial composition away from the diabetic profile, partially overlapping with the Control group, thus suggesting a corrective effect on gut microbial structure (Figure 3F).

At the phylum level, the dominant taxa across all groups included *Bacteroidota*, *Firmicutes*, *Actinobacteriota*, *Proteobacteria*, *Desulfobacterota*, and *Campylobacterota* (Figure 3G). Although the *Firmicutes*-to-*Bacteroidota* (F/B) ratio showed a mild decreasing trend in diabetic rats compared with controls, the difference was not statistically significant. XBL treatment slightly increased the F/B ratio, yet without significant difference among groups, indicating that XBL did not markedly affect the F/B ratio but may help maintain the overall microbial balance (Figure 3H).

Genus-level analysis further revealed significant shifts in several key taxa (Figure 3I). In the model group, the relative abundance of *Prevotella* and *Lachnospiraceae_NK4A136_group* declined sharply, while *Lactobacillus* was markedly elevated. Following XBL treatment, *Prevotella* and *Lachnospiraceae_NK4A136_group* levels increased significantly, while *Lactobacillus* abundance was reduced, realigning the microbial composition toward a eubiotic profile resembling that of healthy controls. These findings indicate that XBL exerts corrective effects on diabetes-induced gut microbial dysbiosis at both phylum and genus levels, thereby contributing to intestinal homeostasis.

### 3.4. Identification of Key Differential Taxa in Response to XBL

**Figure 4:**
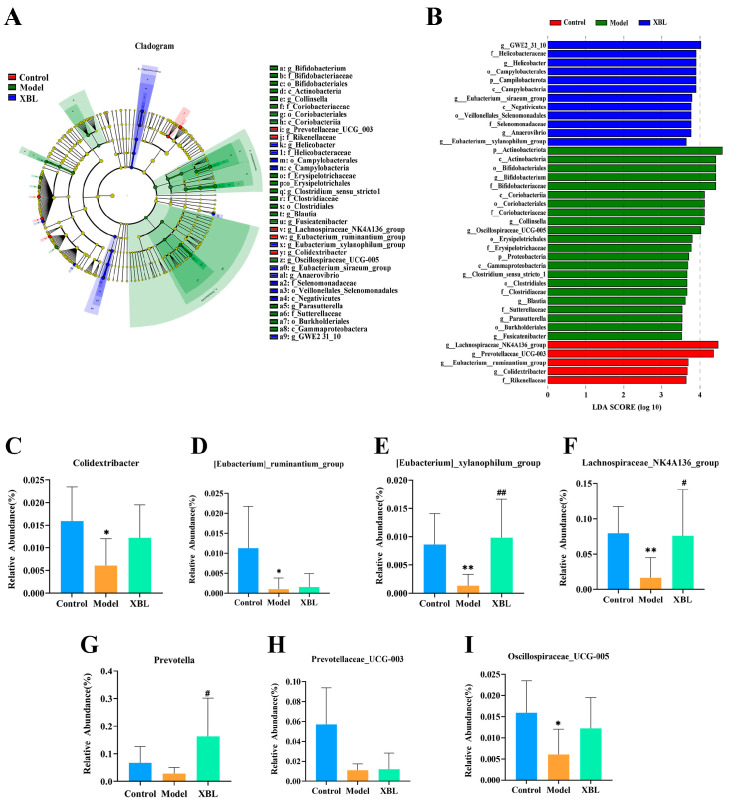
Effects of XBL treatment on gut microbiota composition based on LEfSe analysis in T2DM rats. (**A**) Cladogram showing the taxonomic hierarchy of differentially abundant taxa among the Control, Model, and XBL groups based on linear discriminant analysis effect size (LEfSe). (**B**) Bar plot of taxa with significant differences in abundance identified by LEfSe (LDA score > 3). (**C**–**I**) Relative abundance of key differential genera. Data are presented as mean ± standard deviation (SD) (*n* = 8 per group). Statistical significance: comparisons between Model and Control groups are indicated by * *p* < 0.05, ** *p* < 0.01,; comparisons between XBL and Model groups are indicated by ^#^ *p* < 0.05, ^##^ *p* < 0.01.

To further elucidate how XBL modulates the gut microbial ecosystem under diabetic conditions, we employed linear discriminant analysis effect size (LEfSe) to identify taxa with significant intergroup differences (LDA score > 3.0). This analysis revealed 36 differentially abundant taxa across the three groups, including 16 distinct genera (Figure 4A,B).

Figure 4C–I depict the relative abundance of seven representative genera. Compared to the control group, diabetic rats exhibited significant reductions in *Colidextribacter* (*p* < 0.05), *[Eubacterium]_ruminantium_group* (*p* < 0.05), *[Eubacterium]_xylanophilum_group* (*p* < 0.01), *Lachnospiraceae_NK4A136_group* (*p* < 0.01), and *Oscillospiraceae_UCG-005* (*p* < 0.05). Although *Prevotella* and *Prevotellaceae_UCG-003* also trended downward, these changes were not statistically significant.

XBL treatment significantly increased the abundance of *[Eubacterium]_xylanophilum_group* (*p* < 0.01 vs. model), *Lachnospiraceae_NK4A136_group* (*p* < 0.05), and *Prevotella* (*p* < 0.05), highlighting these taxa as key microbial responders to intervention. Additionally, upward trends were observed in *Colidextribacter* and *Oscillospiraceae_UCG-005*, though without statistical significance. However, no significant recovery was noted for *[Eubacterium]_ruminantium_group* or *Prevotellaceae_UCG-003* following XBL administration. These results identify a subset of beneficial microbial genera that are selectively enriched by XBL and may contribute to its therapeutic effects in T2DM.

### 3.5. XBL Alters the Fecal SCFA Profile in T2DM Rats

To investigate whether the compositional remodeling of the gut microbiota by XBL translates into functional shifts in microbial metabolism, we quantified fecal SCFAs using targeted analysis. SCFAs, particularly acetic, propionic, and butyric acids, are key microbial metabolites implicated in glucose and lipid homeostasis.

**Figure 5:**
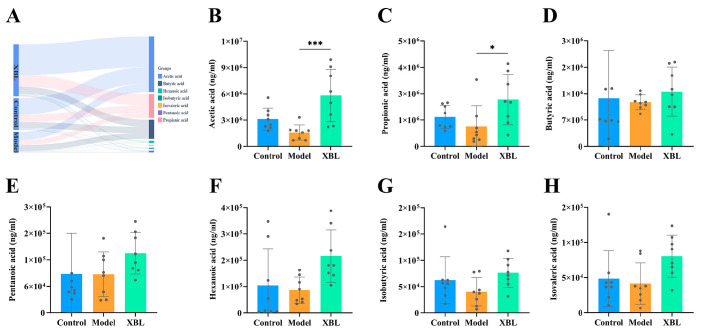
Effects of XBL treatment on short-chain fatty acid (SCFA) production in T2DM rats. (**A**) Sankey diagram illustrating the distribution of total SCFAs among the Control, Model, and XBL groups. (**B**–**H**) Concentrations of individual SCFAs, including acetic acid (**B**), propionic acid (**C**), butyric acid (**D**), pentanoic acid (**E**), hexanoic acid (**F**), isobutyric acid (**G**), and isovaleric acid (**H**). Data are presented as mean ± standard deviation (SD) (*n* = 8 per group). Statistical significance: * *p* < 0.05, *** *p* < 0.001 versus the Model group.

The overall SCFA composition differed markedly among the control, model, and XBL groups (Figure 5A). In diabetic rats, the concentrations of acetic acid (Figure 5B) and propionic acid (Figure 5C) were significantly diminished relative to healthy controls. XBL intervention led to a robust increase in acetic acid (*p* < 0.001) and a moderate rise in propionic acid (*p* < 0.05), restoring these metabolites toward physiological levels. Butyric acid (Figure 5D), although reduced in the model group, exhibited a non-significant upward trend following XBL treatment.

Regarding minor SCFAs, pentanoic acid (Figure 5E) remained stable between the control and model groups but showed a mild, non-significant increase after XBL administration. Hexanoic acid (Figure 5F) was slightly decreased in the model group and modestly elevated by XBL, though without statistical significance. For branched-chain fatty acids (BCFAs), both isobutyric acid (Figure 5G) and isovaleric acid (Figure 5H) were suppressed in T2DM rats but exhibited variable increases post-treatment, again lacking statistical significance.

Taken together, these data suggest that XBL substantially modulates the SCFA profile in T2DM rats, with the most prominent effects observed in acetic and propionic acid production. These SCFAs are known to influence host glucose metabolism, inflammatory tone, and gut barrier integrity, providing mechanistic support for XBL’s metabolic benefits. Importantly, the observed microbial and SCFA changes underscore a functional recovery of the gut microbiota–SCFA axis in response to XBL.

### 3.6. Correlation Analysis Among Gut Microbiota, Host Metabolic Phenotypes, SCFAs, and Fecal Metabolites

To elucidate potential mechanisms underlying the anti-diabetic effects of XBL, Spearman’s correlation analysis was conducted to examine associations among gut microbial taxa, host metabolic parameters, fecal SCFAs, and fecal metabolites. Several genera enriched by XBL—including *[Eubacterium]_ ruminantium_group*, *[Eubacterium]_xylanophilum_group*, *Colidextribacter* and *Lachnospiraceae_NK4A136_group*—exhibited positive correlations with body weight and HDL-C (*p* < 0.05), and negative correlations with fasting blood glucose (FBG) and/or HOMA-IR, indicating potential links to improved glucose and lipid metabolism. Notably, *[Eubacterium]_xylanophilum_group* was also positively associated with insulin levels (*p* < 0.05), while *Lachnospiraceae_NK4A136_group* showed a significant inverse relationship with LDL-C (*p* < 0.01). In contrast, *Oscillospiraceae_UCG-005* was positively correlated with FBG and HOMA-IR (*p* < 0.001) and negatively with HDL-C (*p* < 0.05), suggesting a detrimental metabolic profile. Similarly, *Prevotellaceae_UCG-003* was positively associated with body weight and HDL-C, and negatively with FBG and HOMA-IR (*p* < 0.05), whereas *Prevotella* was inversely correlated with LDL-C (*p* < 0.05). *Muribaculaceae* showed no significant associations with host metabolic indices.

**Figure 6:**
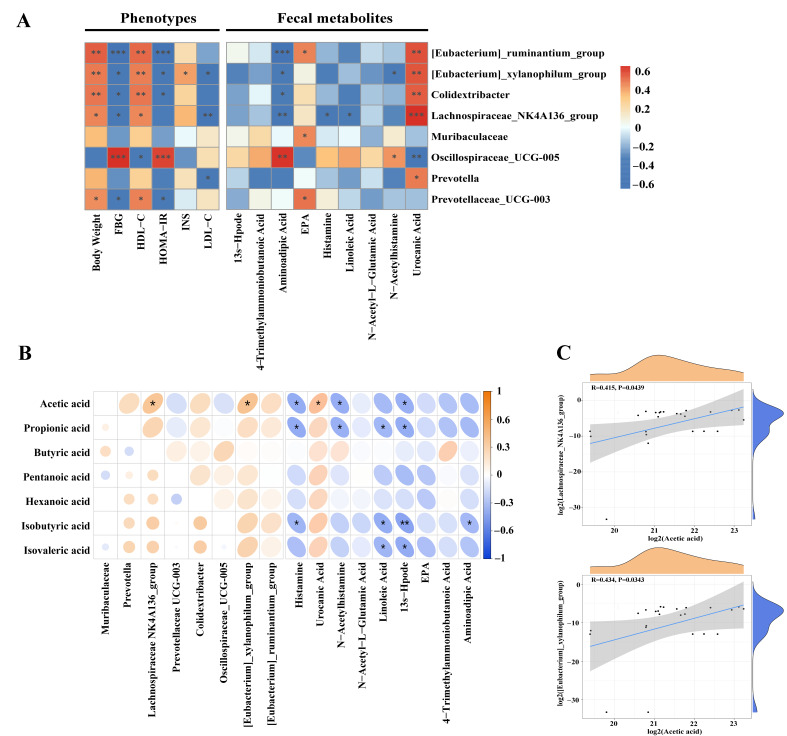
Correlation analysis of gut microbiota, fecal metabolites, SCFAs, and metabolic parameters in T2DM rats. (**A**) Spearman correlation heatmap displaying associations among gut microbiota, fecal metabolites, and serum metabolic parameters, including body weight, FBG, INS, HOMA-IR, LDL-C, and HDL-C. The correlation coefficient (r-value) is indicated by the color gradient, with red representing positive correlations and blue representing negative correlations. (**B**) Spearman correlation matrix showing associations between SCFAs, differential gut microbial genera, and altered fecal metabolites. (**C**) Scatter plots with fitted trend lines illustrating correlations between acetic acid levels and key gut microbial genera based on Spearman’s analysis. Statistical significance: * *p* < 0.05, ** *p* < 0.01, *** *p* < 0.001.

In relation to fecal metabolites, *[Eubacterium]_ruminantium*_*group*, *[Eubacterium]_xylanophilum*_*group*, *Colidextribacter* and *Lachnospiraceae_NK4A136*_*group* were positively correlated with urocanic acid (*p* < 0.01 or stronger), a metabolite previously associated with anti-inflammatory and barrier-protective functions. *[Eubacterium]_ruminantium*_*group* also demonstrated a positive correlation with eicosapentaenoic acid (EPA) (*p* < 0.05) and a strong negative correlation with aminoadipic acid (*p* < 0.001), a metabolite implicated in insulin resistance. Similarly, *Lachnospiraceae_NK4A136_group* was inversely associated with aminoadipic acid (*p* < 0.01), histamine, and linoleic acid (*p* < 0.05), further suggesting beneficial metabolic modulation. Negative correlations with aminoadipic acid were also observed for *[Eubacterium]_xylanophilum*_*group* and *Colidextribacter* (*p* < 0.05), and the former also showed an inverse association with N-acetylhistamine (*p* < 0.05). In contrast, *Oscillospiraceae_UCG-005* was positively correlated with both aminoadipic acid (*p* < 0.01) and N-acetylhistamine (*p* < 0.05), while negatively associated with urocanic acid (*p* < 0.01). Additionally, *Muribaculaceae* and *Prevotellaceae_UCG-003* showed positive correlations with EPA (*p* < 0.05), and *Prevotella* was positively correlated with urocanic acid (*p* < 0.05) (Figure 6A).

To investigate the microbial contribution to SCFA production, correlations were analyzed among SCFAs, microbial genera, and fecal metabolites (Figure 6B). Acetic acid concentrations were significantly and positively correlated with both *Lachnospiraceae_NK4A136*_*group* and *[Eubacterium]_xylanophilum*_*group* (*p* < 0.05), supporting their role in acetate biosynthesis. No other significant microbial associations were observed for SCFA production. Regarding metabolite-SCFA correlations, acetic acid showed a positive association with urocanic acid (*p* < 0.05), and negative associations with histamine, N-acetylhistamine, and 13s-Hpode (*p* < 0.05), suggesting anti-inflammatory and antioxidative links. Propionic acid followed a similar pattern, negatively correlating with histamine, N-acetylhistamine, linoleic acid, and 13s-Hpode (*p* < 0.05). Isobutyric acid was inversely correlated with histamine, linoleic acid (*p* < 0.05), 13s-Hpode (*p* < 0.01), and aminoadipic acid (*p* < 0.05), while isovaleric acid was negatively associated with linoleic acid and 13s-Hpode (*p* < 0.05). No significant correlations were detected for butyric, pentanoic, or hexanoic acids. To further validate the microbial contribution to acetate production, linear regression analysis revealed that acetic acid levels were significantly and positively correlated with *Lachnospiraceae_NK4A136*_*group* (R = 0.415, *p* = 0.0439) and *[Eubacterium]_xylanophilum*_*group* (R = 0.434, *p* = 0.0343), confirming their involvement in SCFA biosynthesis following XBL intervention (Figure 6C). Collectively, these correlations suggest that XBL modulates host metabolism through coordinated regulation of gut microbiota, SCFA production, and metabolite profiles.

## 4. Discussion

This study provides compelling evidence that XBL exerts significant therapeutic effects in T2DM rats, improving glycemic control, enhancing insulin sensitivity, regulating lipid metabolism, and protecting pancreatic and renal tissues from diabetes-induced injury. Unlike earlier investigations that predominantly focused on chemical characterization or in vitro assays, our approach integrates metabolic, histological, and biochemical analyses in vivo, thereby offering a comprehensive evaluation of XBL’s pharmacological efficacy. Notably, XBL treatment led to a significant reduction in fasting blood glucose and HOMA-IR levels, alongside increased insulin secretion, indicative of improved β-cell function and enhanced peripheral insulin sensitivity—both central to T2DM pathogenesis [22].

Histological assessments corroborated these metabolic improvements. XBL preserved the pancreatic islet architecture, reduced vacuolar degeneration, and enlarged islet areas, suggesting protection of β-cell mass and attenuation of islet dysfunction. Renal histopathology further revealed amelioration of tubular dilation, epithelial disarray, and vacuolization, indicating systemic organ-level protection against diabetes-related damage.

In terms of lipid regulation, XBL significantly decreased LDL-C levels and modestly elevated HDL-C, although the latter did not reach statistical significance, potentially due to treatment duration or dosage constraints. These lipid-modulating effects may be mechanistically linked to enhanced fatty acid β-oxidation and cholesterol efflux, consistent with improved lipid metabolic homeostasis [23]. Given that flavonoids and triterpenoids—major constituents of XBL—are known activators of peroxisome proliferator-activated receptor gamma (PPARγ) [24,25,26], and that PPARγ orchestrates both glucose and lipid metabolism [27], its involvement in the observed metabolic benefits is plausible. Furthermore, PPARγ intersects with key signaling cascades such as AMPK and PI3K-Akt [28], both integral to insulin signaling, energy balance, and lipid regulation. These converging pathways likely underlie the systemic metabolic improvements conferred by XBL, reinforcing its promise as a multi-target plant-derived intervention for metabolic disorders.

T2DM is characterized by complex disruptions in glucolipid metabolism, often exacerbated by chronic inflammation, oxidative stress, and dysregulated host–microbiota interactions [29]. Our fecal metabolomics analysis, complemented by KEGG pathway enrichment, revealed that XBL profoundly reshaped the disturbed metabolic landscape associated with T2DM. Specifically, levels of several pro-inflammatory and oxidative metabolites—including 13s-Hpode, histamine, urocanic acid, and linoleic acid—were markedly reduced following XBL treatment. These metabolites are closely linked to endothelial dysfunction and insulin resistance, primarily through the promotion of oxidative injury and inflammatory cascades [30,31,32,33]. For instance, 13s-Hpode, a lipid peroxidation byproduct, has been implicated in vascular damage, while elevated histamine and urocanic acid reflect systemic inflammation and immune dysregulation. Their suppression suggests that XBL contributes to the restoration of redox and inflammatory homeostasis, which may underlie its systemic metabolic benefits.

Although we did not directly quantify inflammatory cytokines, the metabolic profile shifts support the hypothesis that XBL exerts anti-inflammatory effects through the modulation of specific metabolic pathways. Future studies incorporating cytokine profiling and immunological assays would further elucidate this mechanism.

Beyond its impact on inflammatory metabolites, XBL modulated several amino acid metabolism pathways. Notably, the reduction in aminoadipic acid, a lysine catabolite linked to impaired glucose tolerance and insulin resistance [34], suggests alleviation of amino acid–derived metabolic stress. Moreover, XBL influenced histidine and arginine metabolic pathways, which are closely associated with antioxidant defense, nitric oxide production, and endothelial function [35,36,37]. These changes may collectively enhance insulin responsiveness and support vascular integrity, contributing to overall metabolic homeostasis.

Linoleic acid metabolism and unsaturated fatty acid biosynthesis also emerged as significantly altered by XBL. Linoleic acid, an ω-6 polyunsaturated fatty acid and precursor of pro-inflammatory eicosanoids [38], was significantly reduced, implying a potential shift away from lipid peroxidation–driven inflammation. Concurrently, improved regulation of unsaturated fatty acid biosynthesis may support membrane fluidity and insulin receptor signaling, offering additional metabolic benefit through structural and signaling restoration.

Interestingly, levels of N-acetylhistamine—a downstream metabolite of histamine catabolism—were elevated following XBL treatment. Although less extensively studied, N-acetylhistamine is thought to act as a detoxifying agent that neutralizes histamine’s biological activity [39] and may possess anti-inflammatory or neuromodulatory properties via histamine receptor interactions [40]. This elevation could reflect a compensatory response aimed at mitigating histamine-driven metabolic disturbances, highlighting a novel mechanism potentially contributing to XBL’s anti-inflammatory profile.

EPA, a key ω-3 polyunsaturated fatty acid known to reduce inflammation and improve insulin responsiveness via GPR120 activation [41] and regulation of adipokine secretion and macrophage polarization [42], remained low despite treatment. In particular, *Muribaculaceae* and *Prevotellaceae_UCG-003* were positively correlated with fecal EPA levels, suggesting potential microbial–metabolic interactions that are relevant to host lipid metabolism. As direct microbial synthesis of long-chain polyunsaturated fatty acids such as EPA has not been reported in mammalian gut microbiota, these correlations are more likely to reflect microbiota-mediated modulation of host or dietary fatty acid metabolism rather than de novo microbial biosynthesis. This cross-talk between gut microbiota and lipid metabolism may contribute to the beneficial metabolic effects observed following XBL treatment. This incomplete recovery may be attributed to limited biosynthesis, inadequate substrate availability, or competition from ω-6–derived metabolites. These results suggest that while XBL improves overall metabolic balance, adjunct ω-3 supplementation may enhance its therapeutic efficacy.

These findings suggest that, while XBL exerts broad metabolic benefits, adjunct ω-3 supplementation may be required to fully restore anti-inflammatory lipid signaling. The observed results imply that XBL may influence the intestinal metabolic milieu through its interaction with the gut microbial community. To verify this hypothesis, we then examined microbiome data to further explore the underlying mechanisms.

Recent evidence underscores the critical role of gut microbial dysbiosis in the development of T2DM, primarily through disruptions in glucolipid metabolism, chronic low-grade inflammation, and impaired insulin sensitivity [43]. In this study, 16S rRNA gene sequencing revealed a pronounced reduction in microbial richness and diversity in T2DM rats, accompanied by distinct taxonomic shifts. Notably, in contrast to the frequently reported increase in the *Firmicutes*-to-*Bacteroidota* (F/B) ratio in obesity and diabetes [44,45], our model showed a decreasing trend in this ratio, although the difference was not statistically significant. This less common trend has also been observed in other diabetic or high-fat diet-induced models and is considered an alternative manifestation of gut microbial imbalance [46,47]. Additionally, we observed a depletion of beneficial genera such as *Prevotella* and *Lachnospiraceae_NK4A136_group*, further suggesting a disruption of intestinal microbial homeostasis in T2DM rats. These alterations are consistent with previous reports linking gut microbial imbalance to metabolic dysfunction in diabetic models [48,49,50]. Notably, XBL administration reversed many of these aberrations: microbial alpha diversity was restored, community composition shifted toward that of healthy controls, and the F/B ratio normalized. XBL also increased the relative abundance of several health-promoting genera, including *Prevotella*, *Lachnospiraceae_NK4A136_group*, and *[Eubacterium]_xylanophilum_group*—taxa recognized for their capacities to produce short-chain fatty acids (SCFAs), reinforce gut barrier integrity, suppress inflammation, and regulate host metabolism [51].

These compositional changes were functionally reflected in the SCFA profile. Targeted metabolite analysis showed that levels of acetate and propionate were markedly reduced in T2DM rats but significantly restored following XBL treatment. These SCFAs are known to enhance insulin sensitivity, inhibit hepatic gluconeogenesis, and provide energy substrates for colonocytes while modulating immune responses [52]. Although the levels of the branched-chain fatty acids (BCFAs) isobutyric and isovaleric acids were also slightly increased by XBL, these differences did not reach statistical significance, suggesting only a partial enhancement of microbial proteolytic activity. Nevertheless, the overall trend toward the normalization of SCFA and BCFA levels highlights a restructuring of the gut microbiota–metabolite axis in response to XBL.

To further elucidate the mechanisms underlying XBL’s therapeutic effects, we examined correlations among bacterial taxa, metabolic parameters, SCFA levels, and fecal metabolites. Several beneficial genera—including *Lachnospiraceae_NK4A136_group*, *[Eubacterium]_ruminantium_group*, *[Eubacterium]_xylanophilum_group*, *Colidextribacter*, and *Prevotellaceae_UCG-003*—showed positive associations with HDL-C and body weight, and inverse relationships with fasting blood glucose and the insulin resistance index. Specifically, *[Eubacterium]_xylanophilum_group* correlated positively with insulin levels, while *Lachnospiraceae_NK4A136_group* and *Prevotella* were negatively associated with LDL-C. These patterns suggest that XBL-mediated enrichment of select microbial populations contributes to improved glucolipid homeostasis. In contrast, *Oscillospiraceae_UCG-005* was strongly correlated with elevated FBG and HOMA-IR, and negatively associated with HDL-C and urocanic acid, implicating it in T2DM-associated metabolic disturbances.

Microbiota–metabolite associations further support this mechanistic framework. The aforementioned beneficial taxa were positively correlated with urocanic acid—a histidine-derived metabolite with antioxidant and immunomodulatory properties [31]—while *Oscillospiraceae_UCG-005* was linked to higher levels of pro-inflammatory and stress-associated metabolites, including aminoadipic acid and N-acetylhistamine. Notably, *Lachnospiraceae_NK4A136_group* and *[Eubacterium]_xylanophilum_group* were negatively associated with histamine, linoleic acid, and aminoadipic acid, metabolites implicated in oxidative stress and insulin resistance [53,54,55]. These findings suggest that XBL suppresses inflammation-linked microbial metabolism, promoting a more favorable metabolic environment.

SCFA-producing genera also appeared functionally relevant. Acetate levels correlated positively with *Lachnospiraceae_NK4A136_group* and *[Eubacterium]_xylanophilum_group*, indicating their likely contribution to acetate production under XBL treatment. Acetate also showed positive associations with urocanic acid and inverse relationships with pro-inflammatory metabolites such as histamine, N-acetylhistamine, and 13S-Hpode. Similar trends were observed between other SCFAs (e.g., propionate, isobutyrate, isovalerate) and inflammatory metabolites, further implicating SCFAs in the suppression of metabolic stress [56]. While BCFAs showed no statistically significant correlations with specific taxa, their negative associations with stress-related metabolites suggest a broader role in host metabolic regulation [57]. By contrast, butyric, pentanoic, and hexanoic acids did not exhibit significant associations with either microbiota or metabolites, implying a limited role in this experimental context.

Collectively, these findings support a multi-layered mechanistic model in which XBL alleviates T2DM-associated metabolic dysfunction by remodeling the gut microbiota–metabolite network. Through enrichment of beneficial bacterial taxa, enhancement of SCFA production, and suppression of pro-inflammatory metabolic pathways, XBL promotes systemic glucolipid metabolic homeostasis and reduces metabolic stress. These results highlight the therapeutic relevance of gut microbial modulation in dietary intervention strategies for T2DM. Despite these promising findings, several limitations should be acknowledged. The present study was conducted under controlled experimental conditions, and further validation through extended experimental designs and translational studies will be necessary to confirm and expand upon these results.

## 5. Conclusions

This study demonstrates that XBL alleviate T2DM through integrated modulation of host metabolism and gut microbiota. XBL improved glucose and lipid profiles, reduced inflammation, and protected pancreatic and renal tissues. Mechanistically, XBL enhanced microbial diversity, promoted SCFA-producing bacteria, and shifted metabolic pathways such as linoleic acid, histidine, and arginine metabolism toward homeostasis. These findings highlight XBL as a promising functional food ingredient for managing metabolic disorders. Future studies with clinical and human validation are warranted to confirm its therapeutic potential.

## Figures and Tables

**Table 1:** Information on potential biomarkers screened by fecal metabolomics.

Ionization Mode	Metabolites	Mass Error (ppm)	Formula	RT (min)	Measured Mass (*m*/*z*)	Model vs. Control	XBL vs. Model	Pathway
ESI^+^	13s-Hpode	−0.73	C_18_H_32_O_4_	11.01	330.2637	↑	↓ *	Linoleic acid metabolism
	4-Trimethylammoniobutanoic Acid	−0.04	C_7_H_15_NO_2_	0.72	146.1175	↑	↓ *	Lysine degradation
	Urocanic Acid	−0.41	C_6_H_6_N_2_O_2_	0.77	139.0501	↓ ***	↑ *	Histidine metabolism
	Histamine	−0.21	C_5_H_9_N_3_	0.62	112.0869	↑ **	↓ **	
	N-Acetylhistamine	−0.32	C_7_H_11_N_3_O	0.75	154.0974	↑ *	↓ *	
	Linoleic Acid	−0.86	C_18_H_32_O_2_	9.51	281.2473	↑ *	↓ *	Lineolic acids and Biosynthesis of unsaturated fatty acids
	N-Acetyl-L-Glutamic Acid	0.11	C_7_H_11_NO_5_	1.15	212.0530	↑ **	↓	Arginine biosynthesis
ESI^−^	Aminoadipic Acid	−0.93	C_6_H_11_NO_4_	0.68	321.1300	↑ *	↓ *	Lysine degradation
	EPA	−1.31	C_20_H_30_O_2_	12.02	301.2169	↓ ***	↓ **	Biosynthesis of unsaturated fatty acids

* *p* < 0.05, ** *p* < 0.01, *** *p* < 0.001.

## Data Availability

The data that support the findings of this study are available from the corresponding author upon reasonable request.

## Abbreviations

The following abbreviations are used in this manuscript:

FBG	fasting blood glucose
INS	fasting insulin
LDL-C	low-density lipoprotein cholesterol
HDL-C	high-density lipoprotein cholesterol
LC-MS	liquid chromatography-mass spectrometry
SD	Sprague-Dawley
H&E	hematoxylin-eosin
PCA	principal component analysis
OPLS-DA	orthogonal partial least squares discrimination analysis
HMDB	Human Metabolome Database
KEGG	Kyoto Encyclopedia of Genes and Genomes
ASV	amplicon sequence variant
LEfSe	linear discriminant analysis effect size
LDA Score	linear discriminant analysis score
SCFAS	short-chain fatty acids
